# Generalized Liver- and Blood-Derived CD8^+^ T-Cell Impairment in Response to Cytokines in Chronic Hepatitis C Virus Infection

**DOI:** 10.1371/journal.pone.0157055

**Published:** 2016-06-17

**Authors:** Stephanie C. Burke Schinkel, Lorna Carrasco-Medina, Curtis L. Cooper, Angela M. Crawley

**Affiliations:** 1 Department of Biochemistry, Microbiology and Immunology, University of Ottawa, Ottawa, Ontario, Canada; 2 Chronic Disease Program, Ottawa Hospital Research Institute, Ottawa, Ontario, Canada; 3 Department of Epidemiology and Community Medicine, University of Ottawa, Ottawa, Ontario, Canada; 4 Division of Infectious Diseases, Ottawa Hospital-General Campus, Ottawa, Ontario, Canada; 5 Department of Biology, Carleton University, Ottawa, Ontario, Canada; Saint Louis University, UNITED STATES

## Abstract

Generalized CD8^+^ T-cell impairment in chronic hepatitis C virus (HCV) infection and the contribution of liver-infiltrating CD8^+^ T-cells to the immunopathogenesis of this infection remain poorly understood. It is hypothesized that this impairment is partially due to reduced CD8^+^ T-cell activity in response to cytokines such as IL-7, particularly within the liver. To investigate this, the phenotype and cytokine responsiveness of blood- and liver-derived CD8^+^ T-cells from healthy controls and individuals with HCV infection were compared. In blood, IL-7 receptor α (CD127) expression on bulk CD8^+^ T-cells in HCV infection was no different than controls yet was lower on central memory T-cells, and there were fewer naïve cells. IL-7-induced signalling through phosphorylated STAT5 was lower in HCV infection than in controls, and differed between CD8^+^ T-cell subsets. Production of Bcl-2 following IL-7 stimulation was also lower in HCV infection and inversely related to the degree of liver fibrosis. In liver-derived CD8^+^ T-cells, STAT5 activation could not be increased with cytokine stimulation and basal Bcl-2 levels of liver-derived CD8^+^ T-cells were lower than blood-derived counterparts in HCV infection. Therefore, generalized CD8^+^ T-cell impairment in HCV infection is characterized, in part, by impaired IL-7-mediated signalling and survival, independent of CD127 expression. This impairment is more pronounced in the liver and may be associated with an increased potential for apoptosis. This generalized CD8^+^ T-cell impairment represents an important immune dysfunction in chronic HCV infection that may alter patient health.

## Introduction

Acute infection of hepatitis C virus (HCV) is spontaneously cleared in a minority of those infected, and relies on effective virus-specific CD8^+^ T-cell mediated responses [1–4]. Failure to clear the virus is associated with HCV-specific CD8^+^ T-cells with impaired proliferation and cytokine production [5, 6]; a common characteristic of chronic viral infections such as hepatitis B virus (HBV), HIV [7, 8], and HIV-HCV co-infection, as shown by Barrett et al. [9]. This dysfunction is reportedly more pronounced compared to CMV-, EBV-, or influenza-specific cells within the same individual [7, 10, 11]. However, impairment has been observed regardless of antigen specificity in bulk CD8^+^ T-cells, characterized by increased potential for inducible apoptosis and lower basal perforin expression [12, 13]. Hence, CD8^+^ T-cell dysfunction in HCV infection is a generalized phenomenon. While there is no specific clinical immunodeficiency in hepatic viral infections, cirrhosis-associated immune dysfunction syndrome (CAIDS) [14] and increased risk of community-acquired infections such as pneumonia [15, 16] are not uncommon. There is some evidence that progressive liver fibrosis is correlated with impairment of HCV-specific and HCV non-specific CD8^+^ T-cells [17]. Furthermore, bystander CD8^+^ T-cell dysfunction may contribute to a more rapid progression to AIDS in HIV-HCV co-infection compared to HIV mono-infection [18–20].

The mechanisms mediating CD8^+^ T-cell dysfunction in chronic HCV infection are not well understood. Increased IL-10 production by peripheral blood mononuclear cells (PBMC) and IL-10^+^ HCV-specific CD8^+^ T-cells may impair the response [21, 22]. Expression of the inhibitory receptors PD-1 and Tim-3, on both bulk and HCV-specific CD8^+^ T-cells, are associated with reduced proliferation and IFN-γ production [23–26]. Early expression of these receptors on HCV-specific CD8^+^ T-cells can predict progression to chronic infection while high interleukin-7 receptor α (CD127) expression foretells spontaneous clearance and protection [4, 25, 27, 28].

IL-7 is critical for T-cell development and is important for memory cell generation, homeostasis [29–31], as its signalling molecules are directly linked to CD8^+^ T-cell activity (i.e. proliferation, perforin accumulation, Bcl-2 production, and glucose uptake) [32]. In chronic HCV infection, low CD127 expression on HCV-specific CD8^+^ T-cells inversely correlates with viral load, though the expression on bulk CD8^+^ T-cells is similar to controls [33]. The potential role of impaired IL-7 responsiveness in CD8^+^ T-cell dysfunction observed in HCV infection is unknown.

In chronic HCV infection, the dysfunction of CD8^+^ T-cells extends to liver-infiltrating intrahepatic (IH) T-cells. Higher co-expression of PD-1 and Tim-3 on IH-bulk and IH-HCV-specific CD8^+^ T-cells [23, 25, 34], and lower CD127 expression on IH-HCV-specific CD8^+^ T-cells has been observed compared to circulating cells in the same individual [23, 35]. HCV-specific IH-CD8^+^ T-cells have decreased IFN-γ production in response to their cognate antigens compared to other non-HCV-specific memory CD8^+^ T-cells [11], although the function of bulk IH-CD8^+^ T-cells remains largely unknown.

Understanding generalized CD8^+^ T-cell dysfunction in HCV infection will provide insight into the mechanisms establishing chronic infection, progression of liver fibrosis, and other associated immunological impairments. In this report, we tested the hypothesis that bulk circulating and IH-CD8^+^ T-cells in HCV infection have a reduced response to IL-7, and found that CD8^+^ T-cells are phenotypically different with impaired responsiveness to IL-7 detectable among bulk CD8^+^ T-cells.

## Materials and Methods

### Patients

Study participants were healthy, HCV^-^ donors or treatment naïve, chronically infected HCV^+^ individuals (i.e. ≥ 6 months HCV RNA^+^). Age, gender, and ethnicity are summarized in Table 1. Fibrosis scores were determined by fibroscan, and were grouped by fibrosis stage (F0-F2 or F3-F4, with those that are classified as F2-F3 included in the F3-F4 grouping). This study was approved by The Ottawa Health Science Network Research Ethics Board, and written informed consent was obtained from all individuals.

**Table 1:** Baseline Characteristics of Study Participants.

	Controls	HCV^+^ individuals evaluated in functional experiments	HCV^+^ individuals included in whole blood phenotype study
n	51	29	50
Sex (male, female)^a^	M26, F25	M21, F8	M39, F11
Mean age	35.0 ± 10.8	48.9 ± 12.5	49.4 ± 9.9
Ethnicity (%White)	88%	86%	92%
HCV Genotype			
1		22/29	33*/49
2		1/29	6/49
3		6/29	8/49
4		0/29	3*/49
Fibrosis Stage^b^			
0–2		20/28	35/44
2–4		8/28	9/44
Mean HCV RNA (IU/ml)		7.58x106 ± 1.1x107	
Mean ALT^b^		90 ± 58	

^a^ M (male), F (female)

^b^ measured by liver biopsy (Metavir system) or by fibroscan

*One participant with genotype 1 and 4 co-infection

Note: There is no fibrosis or genotype data for some HCV^+^ individuals.

### Isolation and culture of lymphocytes

Peripheral blood mononuclear cells (PBMC) were isolated by Ficoll gradient density centrifugation (Lymphoprep, Stemcell Technologies, Vancouver, Canada), and CD8^+^ T-cells were then isolated by magnetic bead positive selection (Stemcell Technologies, Vancouver, Canada). IH-lymphocytes were isolated from fresh liver biopsies (1mm x 1mm x 3cm) collected from HCV^+^ individuals as part of their routine care and processed by mechanical disruption and enzyme digestion as described previously [36, 37]. Briefly, tissue was cut into small (1mm) pieces and incubated with 500U collagenase IV and 2% FCS in HBSS (Gibco, Life Technologies, Burlington, Ontario, Canada), 50U DNase I (Sigma-Aldrich, Oakville, Ontario, Canada), and 0.6% BSA for 20 minutes at 37°C. Tissue was then manually disrupted with a syringe end and filtered through a 70μm filter (Fisher Scientific, Waltham, MA, USA) to remove undigested tissue. Cells were washed in HBSS and cultured. Both blood-derived CD8^+^ T-cells and IH-lymphocytes were cultured at 1x10^6^/ml in complete RPMI (supplemented with 20% FCS, 1% L-glutamine, and 0.5% penicillin/streptomycin (Gibco, Life Technologies)) at 37°C, 5% CO_2_.

### Flow cytometry and phenotypic analysis of CD8^+^ T-cells

The phenotypes of CD8^+^ T-cells in whole blood (using Optylyse, Beckman Coulter, Marseille, France) or isolated cells were distinguished by flow cytometry using multiple antibodies: CD127-PE (5μl, clone R34.34, AB_131301, Beckman Coulter), CD8-FITC/PeCy5 (5μl, clone HIT8a, AB_395996 and AB_395998), CD45RA-APC/PECy5 (3μl, clone HI100, AB_398468 and AB_395881, BD Pharmingen, BD Bioscience, San Jose, CA, USA) and CCR7-APCCy7 (5μl, clone G043H7, AB_10916390, Biolegend, San Diego, CA, USA). Freshly isolated cells (1x10^5^ lymphocytes per sample) were incubated in 1% BSA-PBS (100μl) for 30 minutes on ice, followed by 2 washes with 1% BSA-PBS, protocol adapted from Nascimbeni and Rehermann [37]. Cell subsets were distinguished as follows: naïve (T_N_, CD45RA^+^CCR7^+^), central memory (T_CM_, CD45RA^-^CCR7^+^), effector memory (T_EM_, CD45RA^-^CCR7^-^), and terminally differentiated effector memory (T_EMRA_, CD45RA^+^CCR7^-^). When analyzing IH-CD8^+^ T-cells, the flow cytometer was calibrated using blood CD8^+^ T-cells to conserve the number of IH-CD8^+^ T-cells available for study, which revealed a higher degree of autofluorescence in IH-CD8^+^ T-cells compared to blood-derived cells, as previously reported by others [38]. All flow cytometry analyses excluded dead cells, on the basis of forward and side scatter profiles, and gates were set using the principle of fluorescence minus one (FMO).

### Measurement of pSTAT5

Isolated blood-derived CD8^+^ T-cells or IH-lymphocytes (0.5x10^6^/ml) were incubated with IL-7 (0.01–10 ng/ml) and/or IL-2 (100 ng/ml) and IL-15 (10 ng/ml, Sigma Aldrich, St. Louis, MO, USA) for 15 minutes at 37°C and phosphorylation of STAT5 (pSTAT5) was measured by flow cytometry, as described previously using the anti-pSTAT5 pY694 alexafluor 488 antibody (5μl/100μl cells, clone 47/STAT5(pY694), AB_399881, BD Phosflow, BD Bioscience) with fixation in 4% paraformaldehyde and permeabilization in 100% cold methanol [32] (average age of controls was 39 ± 13, HCV^+^ individuals was 43 ± 12). The expression level of pSTAT5 in CD8^+^ T-cell subsets was determined after cells were stained for phenotypic markers using CD45RA-APC and CCR7 antibodies (average age of controls was 35 ± 11, HCV^+^ individuals was 56 ± 10). To distinguish CD8^+^ T-cells from other IH-lymphocytes, 5μl CD8-PeCy5 was added with the pSTAT5 antibody. The autofluorescence of IH-lymphocytes is higher than blood-derived cells, and this was taken into account during data analysis [38].

### Proliferation of CD8^+^ T-cells

Isolated CD8^+^ T-cells were labeled with carboxyfluoresceinsuccinimidyl ester (CFSE, 8μM, Cell Trace CFSE Cell Proliferation Kit, Molecular Probes, Life technologies) and cultured with IL-7 (10ng/ml) and a suboptimal concentration of the T-cell mitogen phytohaemagglutinin (PHA, 0.2ug/ml, Sigma Aldrich) for 5 days, as described previously [39] (average age of controls was 31 ± 11, HCV^+^ individuals was 52 ± 02). Colchicine (100ng/ml, Sigma Aldrich) was used a negative control. Proliferation (CFSE dilution) was determined by flow cytometry.

### Measurement of Bcl-2 Expression

Expression of Bcl-2 in CD8^+^ T-cells was determined after overnight rest of isolated cells and after incubation with IL-7 (0.01-10ng/ml) for 48 hours. Specifically, cells were analysed by flow cytometry using an anti-Bcl-2 FITC (5μl/100μl cells, clone Bcl-2/100, AB_396382) antibody, and an IgG1-FITC isotype control (5μl/100μl cells, clone MOPC-21, BD Pharmingen) as described previously [32] with fixation in 4% paraformaldehyde and permeabilization in 1% saponin (Sigma Aldrich) (average age of controls was 32 ± 11, HCV^+^ individuals was 46 ± 12). To distinguish CD8^+^ T-cells from other IH-lymphocytes, 5μl CD8-PeCy5 was added before fixation in 1% BSA-PBS (100μl) for 20 minutes at room temperature. The autofluorescence of IH-lymphocytes relative to blood cells was taken into account during data analysis [38].

### Analysis and Statistics

Flow cytometry was completed using an FC500 Beckman Coulter flow cytometer followed by analysis using FCS Express Research Edition 4.0 (De Novo Software, Los Angeles, CA, USA). Graphs and statistics were generated using GraphPad Prism 5.0 Software (San Diego, CA, USA). Where necessary, statistical analyses included two-way, unpaired Student’s *t*-test, one-way ANOVA with Dunnett post-test, and/or non-linear regression (p ≤ 0.05), and data are presented in text and graphical form as means ± standard deviation.

## Results

### Blood-derived CD8^+^ T-cell subsets differ between HCV^-^ controls and HCV-infected individuals

**Fig 1:**
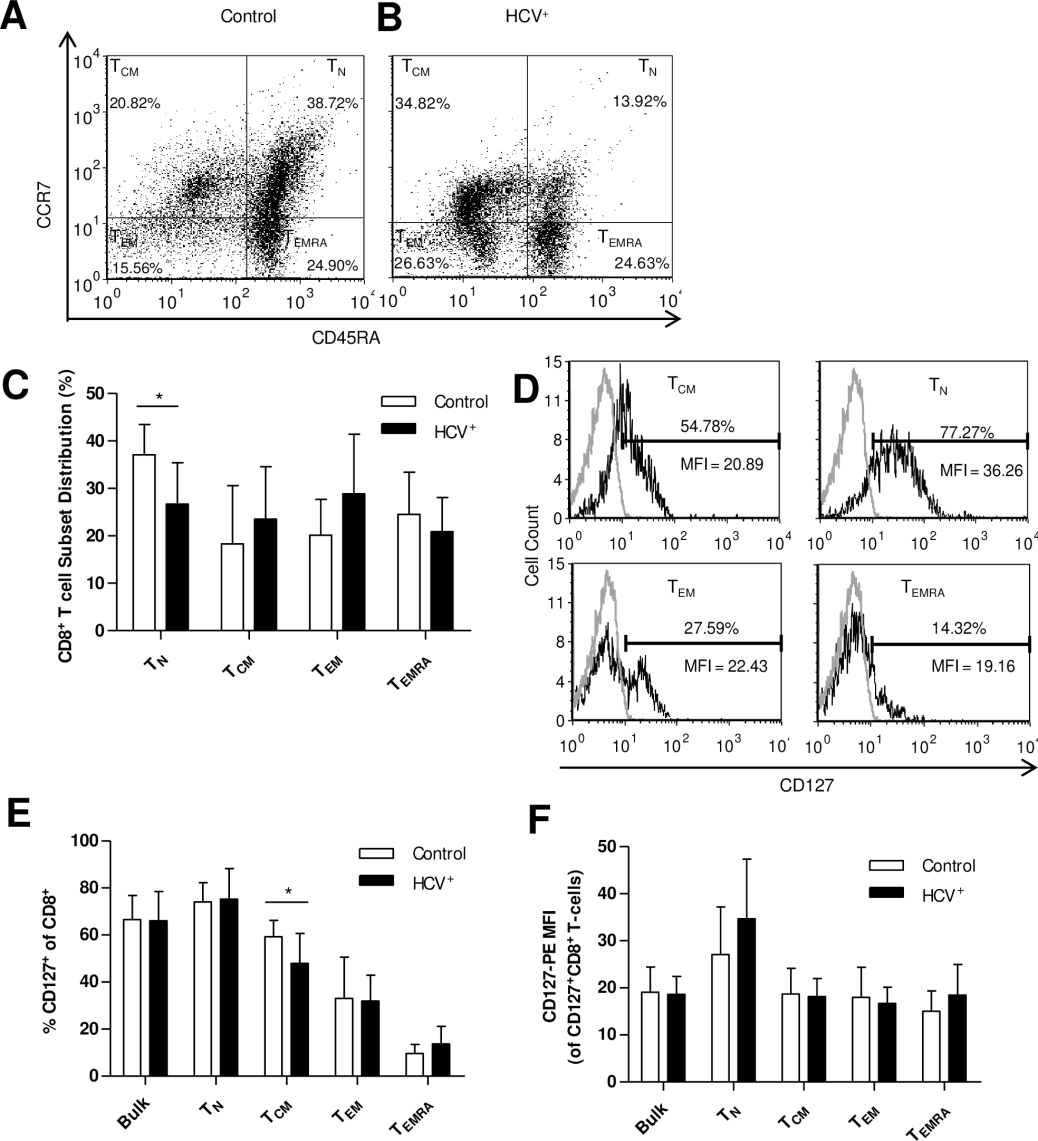
HCV^+^ individuals have fewer blood-derived naïve CD8^+^ T-cells and a lower expression of CD127 on central memory cells than HCV^-^ controls. CD8^+^ T-cell subset distribution was determined by CD45RA and CCR7 staining in (A) controls (n = 10) and (B) HCV infection (n = 12). (C) Subset distribution data for controls and HCV-infected individuals are graphically represented as means (T_N_ p = 0.006). (D) The expression of CD127 was measured on blood-derived bulk CD8^+^T-cells (control n = 30, HCV^+^ n = 50) and their subsets (control n = 10, HCV^+^ n = 12) and presented as (E) percentage (T_CM_ p = 0.02, unpaired Student’s *t*-test) and (F) mean fluorescence intensity of CD127 expressing cells (error bars represent ±S.D.).

The distribution of blood-derived CD8^+^ T-cell subsets was evaluated to detect any inherent differences in chronic HCV infection. PBMCs were stained with anti-CD8 antibody, and subsets distinguished with anti-CD45RA and anti-CCR7 antibodies, as established by Sallusto et al [40]. Blood-derived CD8^+^ T-cell subset distribution in HCV^-^ individuals was as follows (mean ± SD): T_N_ (37.1% ± 2.0) > T_EMRA_ (24.5% ± 2.8) > T_EM_ (20.12% ± 2.4) > T_CM_ (18.3% ± 3.9) (Fig 1A), and is consistent with previous reports [31, 41]. In chronic HCV infection, the ranking of subsets was subtly different: T_EM_ (28.9% ± 3.6) > T_N_ CD8^+^ T-cells (26.7% ± 2.5) > T_CM_ (23.5% ± 3.2) > T_EMRA_ (20.9% ± 2.1) (Fig 1B). There were no significant differences in the proportions of T_EM_, T_CM_ and T_EMRA_ cells compared to controls. However, the proportion of T_N_ cells was significantly lower in HCV^+^ individuals (p = 0.006 by Student’s *t*-test, Fig 1C and 1D). There was no detectable association between CD8^+^ T-cell subset distribution and fibrosis stage or HCV genotype. However, HCV^+^ individuals in the older age group (≥58 years of age, ≈ 75% percentile of individuals tested) did have an increased proportion of T_EMRA_ cells. In all other experiments, within HCV^+^ individuals tested, age was not associated with differences in the measured result.

### The expression of CD127 on bulk blood-derived CD8^+^ T-cells does not differ, though is lower on T_CM_ cells, in HCV infection

To determine if the degree of CD127 receptor expression could contribute to IL-7 responsiveness in subsequent experiments, receptor expression was assessed. In bulk blood-derived CD8^+^ T-cells, there was no difference in percentage of CD127 expression between HCV^-^ controls and HCV-infected individuals (66.7% ± 1.9 and 66.1% ± 1.8, respectively, Fig 1E), nor in the degree of CD127 expressed on these cells (MFI, Fig 1F). The proportion of CD8^+^ T-cells expressing CD127 varied by subset; in controls, it was as follows (mean ± SD): T_N_ (74.1% ± 2.6) > T_CM_ (59.3% ± 2.2) > T_EM_ (33.1% ± 5.5) > T_EMRA_ (9.6%± 1.2) (Fig 1D and 1E). This hierarchical pattern of CD127 expression was similar in HCV infection for 3 subsets: T_N_ (75.3% ± 3.8) > T_EM_ (32.0% ± 3.2) > T_EMRA_ (13.7% ± 2.2). There were significantly fewer T_CM_ cells expressing CD127 in HCV infection (48.0% ± 3.7) compared to controls (p = 0.02 by Student’s *t*-test, Fig 1E. The intensity of CD127 expression on subsets expressing CD127 was the highest in T_N_ cells, with similar intensity in the other 3 subsets, for both experimental groups (Fig 1F). Levels of CD127 expression were not associated with age, fibrosis stage or HCV genotype among HCV^+^ individuals.

### IL-7 signalling through STAT5 is impaired in blood-derived CD8^+^ T-cells in HCV infection

**Fig 2:**
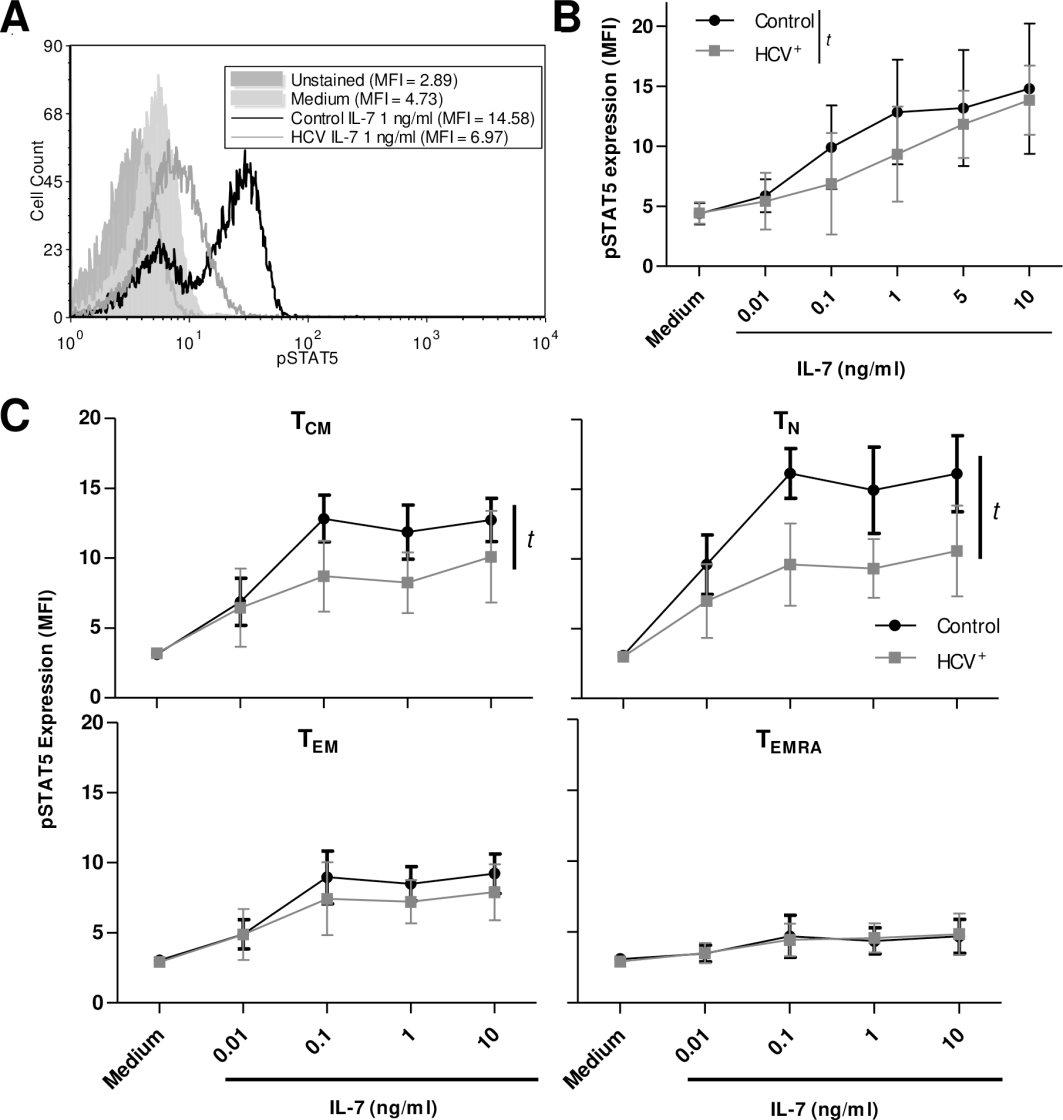
IL-7-induced signaling of blood-derived CD8^+^ T-cells is impaired in HCV infection. (A) Phosphorylation of STAT5 was measured as mean fluorescence intensity (MFI) as shown in a representative histogram. (B) The expression of pSTAT5 was significantly increased by increasing concentrations of IL-7 (0.01–10 ng/ml) in blood-derived CD8^+^ T-cells from controls (p < 0.001, n = 10) or chronically infected HCV^+^ individuals (p = 0.003, n = 9) is summarized, as assessed by ANOVA, yet responses of the latter group were significantly less pronounced than controls (*t*: p = 0.005, non-linear regression analysis). (C) The expression of pSTAT5 in CD8^+^ T-cell subsets were distinguished by CD45RA and CCR7 expression, with significance in T_CM_ and T_N_ subsets (*t*: p<0.0001 for each subset, non-linear regression, control n = 7, HCV^+^ n = 5). Error bars in the graphs represent ± S.D.

To investigate activation of the Jak/STAT pathway in response to IL-7 in health and chronic HCV infection, the phosphorylation of STAT5 (pSTAT5) was evaluated in CD8^+^ T-cells. Phosphorylation of STAT5 in response to IL-7 (0.01-10ng/ml) occurred in a dose dependent manner in CD8^+^ T-cells isolated from HCV^-^ controls and HCV-infected individuals (one-way ANOVA p<0.0001 and p = 0.0003, respectively), as expected [32, 42] (Fig 2A and 2B). Upon IL-7 stimulation, the minimum, and physiological, concentration of IL-7 required for a significant increase in pSTAT5 was lower for controls (0.1ng/ml) than in HCV infection (1ng/ml) (p≤ 0.05, Dunnett post-test). There were no associations with pSTAT5 production and age detected. Since there were no individuals with fibrosis scores >2 among those tested in this assay, it was not possible to analyze any association between the degree of STAT5 activation and liver fibrosis. Overall, across these dose responses, there was less STAT5 activation in IL-7-stimulated CD8^+^ T-cells from HCV-infected individuals compared to controls (non-linear regression, p = 0.005).

The degree of pSTAT5 expression by IL-7-stimulated CD8^+^ subsets in controls (Fig 2C) corresponded with their stratified expression of CD127 (Fig 1E). In control individuals, T_N_ cells phosphorylated STAT5 to the greatest extent, followed by T_CM_ and T_EM_ cells, while T_EMRA_ cells did not phosphorylate STAT5. In HCV infection, IL-7 stimulation activated STAT5 in T_N_ and T_CM_ cells but was significantly lower than in controls (p<0.0001, non-linear regression). Unlike in bulk CD8^+^ T-cells, the level of pSTAT5 with the highest concentration of IL-7 (10ng/ml) was lower in HCV infection than controls. Unfortunately, the relation between the degree of fibrosis and IL-7-induced pSTAT5 levels could not be examined as only 2 individuals in the study of bulk CD8^+^ T-cells and 1 individual in the CD8^+^ T-cell subset studies had fibrosis scores > F2.

### Proliferation is not impaired but blood-derived CD8^+^ T-cells express less Bcl-2 in response to IL-7 in HCV infection

**Fig 3:**
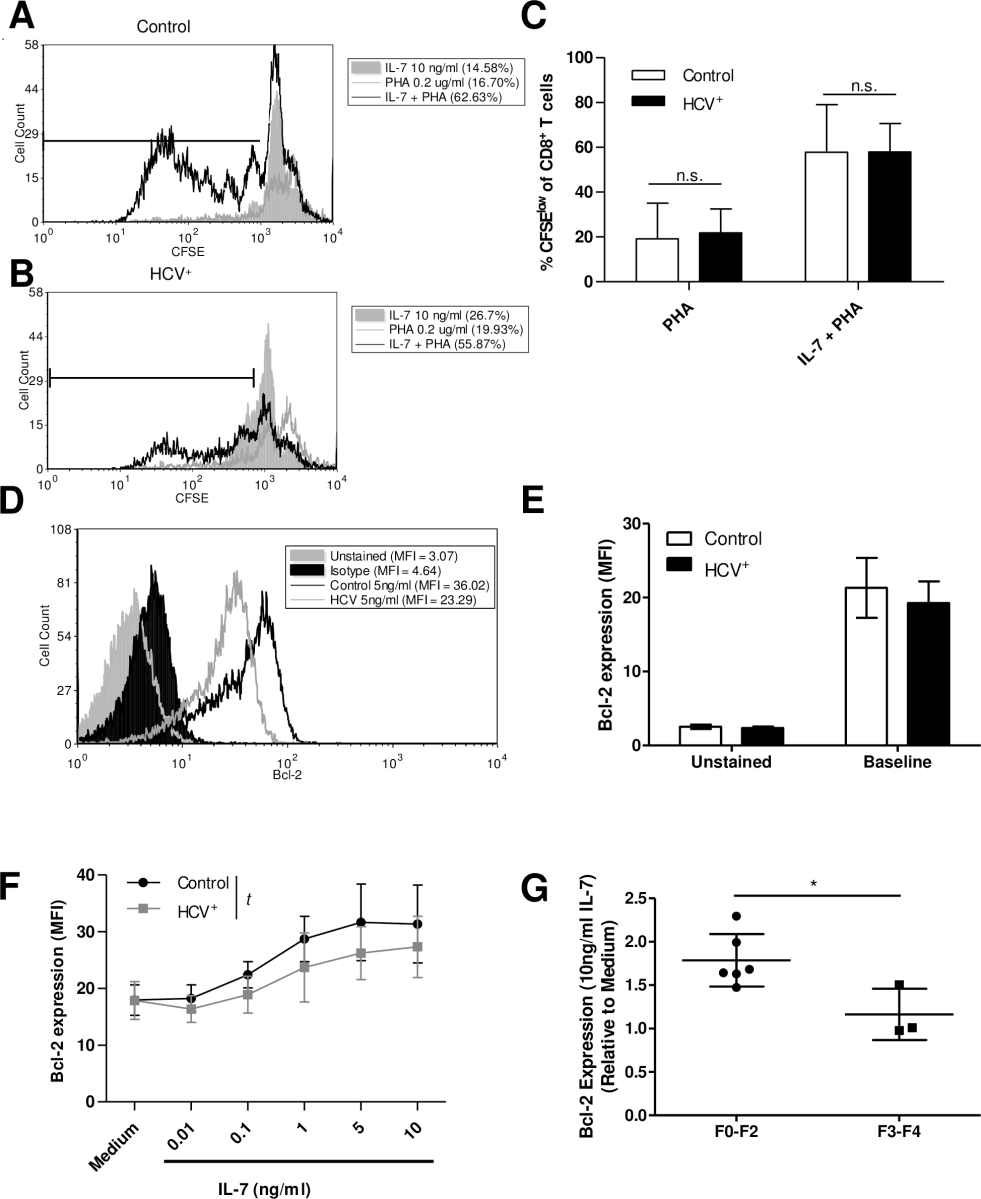
IL-7-induced proliferation is not impaired while production of Bcl-2 is reduced in blood-derived CD8^+^ T-cells from HCV^+^ individuals. Cell proliferation in response to IL-7 (10ng/ml) and/or PHA (0.2mg/ml) was measured as CFSE dilution of CD8^+^ T-cells from (A) HCV^-^ (n = 8) and (B) HCV^+^ individuals (n = 8), with markers indicating the proportion (%) of CFSE^low^ (dividing) cells. (C) Proliferation of isolated CD8^+^ T-cells induced by IL-7 + suboptimal PHA was significantly increased in both groups (control p = 0.0001 and HCV^+^ p<0.0001, unpaired Student’s *t*-test), yet there was no difference between these groups (n.s. = not significant). (D) Bcl-2 expression of blood-derived CD8^+^ T-cells was measured. (E) Bcl-2 expression (MFI) of unstimulated CD8^+^ T-cells is summarized (control n = 4, HCV^+^ n = 5) and (F) Bcl-2 production in response to IL-7 after 48 hours is summarized as MFI (*t* p = 0.0006, non-linear regression, control n = 8, HCV^+^ n = 9). (G) The IL-7-induced expression of Bcl-2 in blood-derived CD8^+^ T-cells from HCV^+^ individuals with low fibrosis (F0-F2) was compared to that of high fibrosis (F3-F4). Values are expressed relative to medium alone (* p = 0.02, unpaired Student’s *t*-test, error bars represent ±S.D.).

To quantify IL-7-mediated proliferation of blood-derived CD8^+^ T-cells, CFSE-labelled cells were stimulated with suboptimal amounts of T-cell mitogen (PHA, 0.2 ug/ml), since T-cell activation is required to enable human T-cells to proliferate in response to IL-7 [43]. Cells were cultured with a dose of IL-7 known to induce detectible cell division (10ng/ml) [42]. There was minimal proliferation of cells following culture with IL-7 or PHA alone, while IL-7 + PHA induced multiple cell divisions (%CFSE^low^ cells) (Fig 3A and 3B). However, there was no difference between the IL-7 + PHA mediated proliferation of CD8^+^ T-cells isolated from HCV^-^ controls (n = 8, 57.9% ± 7.5) and HCV^+^ individuals (n = 8, 58.0% ± 4.5), nor was there a difference in their proliferation in response to PHA alone (19.2% ± 5.6 and 21.9% ± 3.8, respectively, Fig 3C). No association between age and proliferation was detected among HCV^+^ individuals, and a correlation analysis of fibrosis score with proliferation was not possible as only 2 out of 9 individuals tested here had scores greater than F2.

To determine the potential for cell survival, the expression of anti-apoptotic Bcl-2 was measured. The *ex vivo* expression of Bcl-2 of blood-derived CD8^+^ T-cells from controls and HCV infection were similar (Fig 3E). Culture of CD8^+^ T-cells with IL-7 (0.01-10ng/ml) increased Bcl-2 levels in a dose-dependent manner in cells isolated from either controls or HCV-infected individuals (Fig 3D and 3F) (one-way ANOVA p<0.0001 for each group). However, despite similar Bcl-2 basal expression across the groups after overnight culture, the magnitude of increase in Bcl-2 expression in response to increasing concentrations of IL-7 was lower in HCV infection (p = 0.0006 by non-linear regression). Similar to STAT5 activation (Fig 2B), Bcl-2 levels were not significantly increased in CD8^+^ T-cells of HCV-infected individuals when stimulated with less than 1ng/ml of IL-7, while cells from controls produced significantly more Bcl-2 with a log lower concentration of IL-7 (0.1ng/ml, p≤ 0.05, Dunnett post-test).

Of the individuals tested in these Bcl-2 assays, 3 had fibrosis scores of F3-F4 and 6 had scores of F0-F2. Individuals with higher scores of liver fibrosis (i.e. F3-F4 scores) produced significantly less Bcl-2 in response to IL-7 (10ng/ml) compared to those with lower degrees of fibrosis (i.e. F0-F2) (p = 0.02, unpaired Student’s *t*-test) (Fig 3G). Therefore, while proliferation of CD8^+^ T-cells in response to IL-7 was not different between controls and HCV-infected individuals, there was a significant reduction in IL-7-induced Bcl-2 production that was inversely associated with the extent of fibrosis.

### Intrahepatic CD8^+^ T-cells have an increased proportion of T_EM_ cells compared to blood-derived cells in HCV infection, while CD127 expression remains unchanged

**Fig 4:**
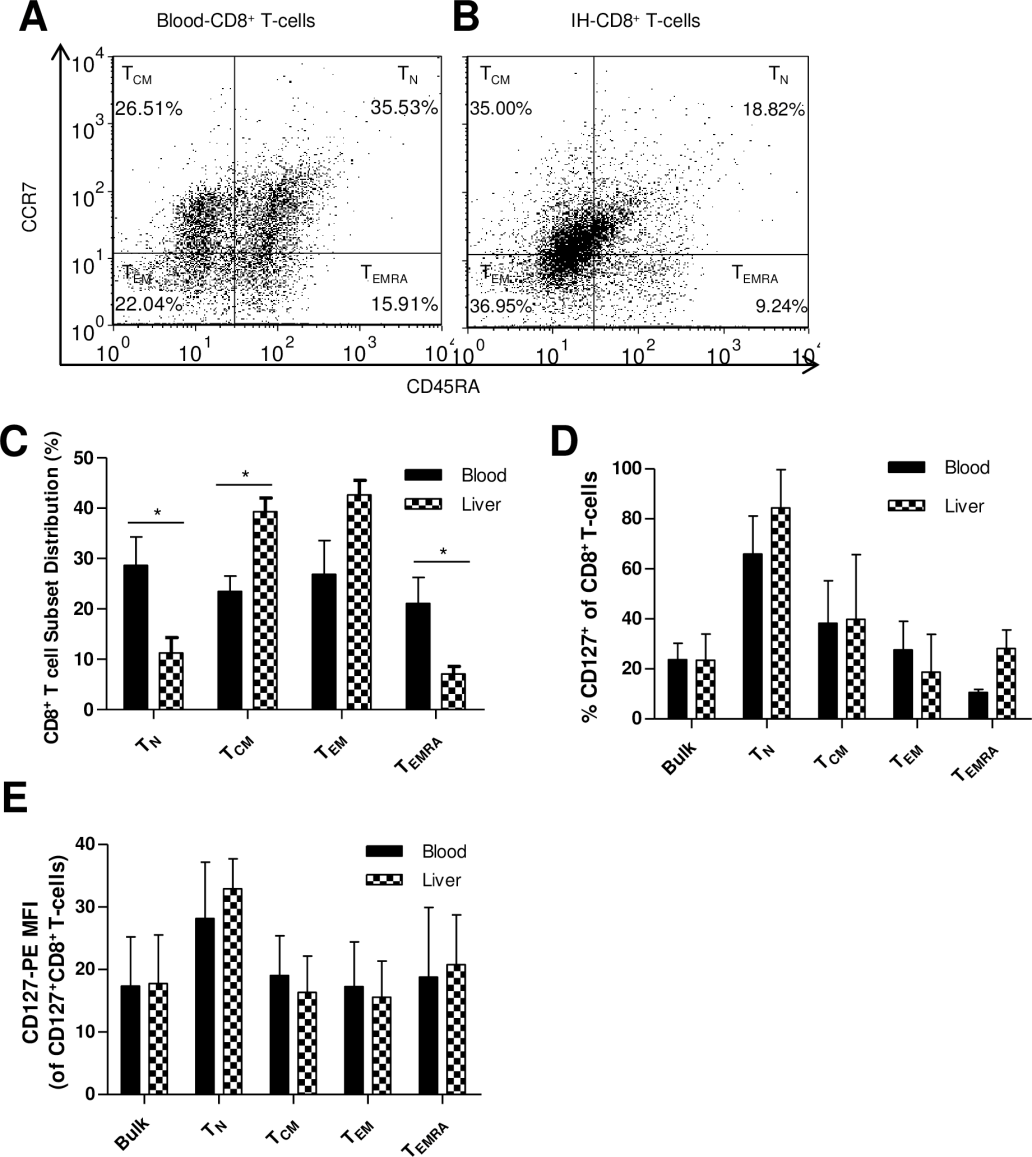
The proportion of CD8^+^ T _CM_ cells is increased, while T_N_ and T_EMRA_ cells are decreased in the liver in HCV infection and CD127 expression does not differ between IH- and blood-derived CD8^+^ T-cells. Representative dot plots of subset distribution are shown for (A) Blood-derived and individually matched (B) IH-CD8^+^ T-cell subsets, as analysed by flow cytometry which distinguished between subsets on the basis of CD45RA and CCR7 expression. (C) The means of these observations are summarized in a bar graph (* T_N_ p = 0.03, T_CM_ p = 0.01, T_EMRA_ p = 0.03, unpaired Student’s *t*-test, n = 3, error bars represent ± S.D.). Membrane CD127 expression on bulk and CD8^+^ T-cells subsets did not differ between locations, as measured by (D) percentage expression nor (E) intensity of expression (MFI).

IH-lymphocytes were isolated from 4 separate liver biopsy samples obtained from HCV^+^ individuals and the proportions of CD8^+^ T-cell subsets were simultaneously compared to blood-derived CD8^+^ T-cells from the same individuals. The phenotypic distribution of blood CD8^+^ T-cells in these individuals was relatively similar to proportions reported in Fig 1 (Fig 4A and 4B). The phenotype of IH-CD8^+^ T-cells differed significantly compared to blood CD8^+^ T-cells, with a higher proportion sharing cell markers with T_CM_ (39.3% ± 5.3 vs. 23.5% ± 3.1, p = 0.03, unpaired Student’s *t*-test), and a lower proportion expressing T_N_ (11.2% ± 6.2 vs. 28.6% ± 5.6, p = 0.01) and T_EMRA_ (7.08% ± 3.0 vs. 21.07% ± 5.2, p = 0.03) surface proteins. There was also a trend of more cells lacking CCR7 and CD45RA surface proteins, and appearing to be T_EM_ than in blood CD8^+^ T-cells (42.64% ± 5.7 vs. 26.8% ± 6.7, p = 0.06) (Fig 4C). The CD8^+^ T-cells found in the liver appear to have a single, memory-like phenotype.

The level of CD127 expression was equivalent between blood-derived and IH-CD8^+^ T-cells (27.8% ± 3.8 and 23.6% ± 6.0, respectively) (Fig 4D). Similarly, CD127 expression among CD8^+^ T-cell subsets was the same between controls and HCV-infected individuals, and followed the same hierarchical pattern: T_N_ (66.0% ± 8.8 and 81.3% ± 13.9) > T_CM_ (38.2% ± 17.1 and 42.6% ± 21.8) > T_EM_ (27.6% ± 6.6 and 22.3% ± 14.3) ≈ T_EMRA_ (10.7% ± 0.7 and 23.5% ± 11.0) (Fig 4D). The intensity of CD127 expression was similar to percentage expression, with T_N_ cells expressing the most CD127 in both blood and liver (Fig 4E).

Observational data from 2 individuals has indicated that IH-CD8^+^ T-cells expressed high basal levels of pSTAT5 compared to blood CD8^+^ T-cells, and IL-7 and other common γ chain (γ_c_) cytokines (e.g. IL-2 and IL-15) did not further increase pSTAT5 (S1A Fig). The degree of basal Bcl-2 expression after overnight culture was lower in IH-CD8^+^ T-cells compared to blood-derived CD8^+^ T-cells in HCV mono-infection (S1B Fig). Further investigation of these novel findings is not possible given recent limitations in access to liver biopsies for HCV-infected patients at The Ottawa Hospital where standard of care now mandates the use of ultrasound diagnostics (i.e. Fibroscan).

## Discussion

CD8^+^ T-cells isolated from individuals with HCV infection exhibit significantly impaired responsiveness to IL-7, independent of CD127 expression, and this was widespread, as detected in bulk blood-derived and IH-CD8^+^ T-cells. Impaired HCV-specific CD8^+^ T-cell has been well described [7, 10, 21, 22, 26, 28, 35], while bystander CD8^+^ T-cell dysfunction has been less well understood [12, 13]. Impaired response of bulk CD8^+^ T-cells to cytokine stimulation suggests broad CD8^+^ T-cell dysfunction in HCV infection, which this study has simultaneously observed in both circulating and liver-infiltrating CD8^+^ T-cells. This was a challenging study, as the relatively low number of IH-CD8^+^ T-cells in a liver biopsy of an HCV-infected individual limited the breadth of these investigations. In addition, the recent increased use of elastography diagnostics (i.e. fibroscan), instead of liver biopsies, has significantly reduced the availability of this tissue for this research. Despite these challenges, our small data set from this tissue provides invaluable insights into the state of IH-CD8^+^ T-cells in HCV infection, which complements our findings in circulating cells.

We found no role for CD127 expression in the CD8^+^ T-cell dysfunction observed here. In HCV infection, there were fewer T_N_ blood-derived CD8^+^ T-cells compared to healthy controls (Fig 1C), similar to a previous report [44], but no CD127 expression differences were observed except in the T_CM_ cell subset (Fig 1D and 1F). This suggests an inherent cell deficiency similar to that of dysfunctional CD8^+^CD127^+^ T-cells in HIV-infected individuals (e.g. reduced IL-7 signalling, survival and proliferation) [42, 45], contrasted in part by the marked decrease of CD127 expression on bulk CD8^+^ T-cells in HIV infection [46–49]. In addition, receptor expression was not associated with age in HCV infection, unlike in HIV infection [50]. It is not likely that other elements of the IL-7 receptor complex contribute to this impairment, as IL-7 does not alter CD132 expression and chronic viral infection can increase the proportion of CD132 expression [51, 52].

A previous study where increased susceptibility of CD8^+^ T-cells to apoptosis was noted [12] supports our finding that IL-7 did not increase Bcl-2 expression of CD8^+^ T-cells in HCV infection to the same level as in controls (Fig 3F). The decreased expression of Bcl-2 did not translate into decreased CD8^+^ T-cells isolated in HCV infection, as the percentage of CD8^+^ cells among PBMC for control and HCV infection were similar (17.1 ± 7.4 and 17.5 ± 6.0, respectively). This decreased Bcl-2 finding also confirms our previous report, and that of others, that Bcl-2 production is dependent on STAT5 activation [32, 53], which was also found to be lower in IL-7-stimulated cells from HCV-infected individuals compared to controls. The lower level of STAT5 activation with IL-7 stimulation observed in HCV infection was most evident in blood-derived T_CM_ and T_N_ cells, subsets with the highest level of STAT5 activation (Fig 2C). This was not associated with lower CD127 expression as only T_CM_ cells had a lower CD127 (Fig 1F). The proliferation of CD8^+^ T-cells induced by IL-7 was similar between controls and HCV infection (Fig 3C), unlike HIV infection [42]. The activation of STAT5 is also associated with T-cell proliferation, however activation of the Akt pathway may have compensated for the inadequacies of STAT5 signaling in this instance, although our experimental efforts could not reliably assess Akt activity in these samples [54].

In viral hepatitis (HCV and HBV), IH-CD8^+^ T-cells frequently have an activated phenotype (i.e. decreased CD28 and increased IFN-γ expression) [55], similar to the T_CM_/T_EM_ type phenotype of the IH-CD8^+^ T-cells observed here (Fig 4B). Our further analysis of cytokine signaling and survival potential was limited to observational results of two individuals of blood and liver matched samples (S1 Fig) due to the recent lack of access to liver biopsies from HCV-infected individuals. The high level of basal STAT5 activation observed (S1A Fig) may be due to cytokine secretion by hepatocytes, a known source of IL-7 [56]. As a tertiary lymphoid organ, liver dendritic cells, hepatocytes, and Kupffer cells expressing co-stimulatory molecules can also activate CD8^+^ T-cells in inflammatory conditions [57–60]. However, T-cells activated in the liver are more prone to apoptosis and produce less IFN-γ and IL-2 [58, 61, 62]. This may contribute to the reduced Bcl-2 expression observed here in IH-CD8^+^ T-cells in HCV infection (S1B Fig), and may partially explain the increased susceptibility of IH-CD8^+^ T-cells to apoptosis. The effect of such decreases in Bcl-2 expression on the life span CD8^+^ T-cells and their function *in vivo* is not known as decreases in Bcl-2 levels do not guarantee apoptosis, and would have to be assessed directly. However, if future access to liver sample permits, further analysis of blood-derived vs. IH-CD8^+^ T-cells would be required to confirm these findings.

Higher stages of fibrosis (F3-F4) were associated with lower IL-7-mediated Bcl-2 expression by blood-derived CD8^+^ T-cells (Fig 3G). No other associations were detected between CD8^+^ T-cell activities and fibrosis stage, although the selection of study subjects was not stratified to examine immune function associations with liver damage. Few associations between fibrosis and CD8^+^ T-cell function in HCV infection have been described; fibrosis has been associated with increased infiltration of CD8^+^ T-cells as well as increased CD8^+^ T-cell apoptosis in pediatric and adult livers in HCV infection [63, 64]. Whether the impairment observed is due to chronic liver disease, or HCV specifically, was not determined. The inclusion of controls with non-HCV chronic liver disease may have offered some insight in this regard. However, in alcoholic liver disease, the extent of liver damage has been implicated as a potential contributor to reduced T-cell responses, in the absence of infection [65]. Similarly, cirrhosis-associated immune dysfunction syndrome includes states of immune depression, including CD8^+^ T-cell exhaustion and senescence that is dependent on the severity and etiology of the liver disease [14, 66]. Nevertheless, NASH, alcoholic liver disease (ALD) and HBV/HCV infections recruit many CD8^+^ T-cells to the liver regardless of their antigen specificity. Activation of these cells in this organ in these diseases is associated with increased pro-inflammatory and pro-fibrogenic cytokine production [22, 67–70]. In HCV infection, increased CD8^+^ T-cell infiltrates in fibrosis is also associated with elevated levels of liver CD8^+^ T-cell apoptosis [63, 64], suggesting a potential common feature of liver disease.

Potential contributors to the suppression of T-cell activity and cytokine response include direct viral effects (plasma HCV core protein) or changes in the expression of cytokine signalling regulatory proteins (e.g. suppressors of cytokine signalling) [8, 71, 72]. In addition, the duration of the infection, host genetics, smoking or age were not evaluated here. A sizeable proportion of the CD8^+^ T-cell pool is comprised of CMV-specific CD8^+^ T-cells in healthy individuals, and numbers increase with age (>60yrs), found principally as a late differentiation phenotype (e.g. T_EMRA_) [73–75]. While CMV-specific T-cell responses are known to be strong, their proliferative potential *in vitro* is poor and their effector functions are weak following solid organ transplantation and in immunocompromised individuals [76, 77]. In this study, the HCV-infected individuals tested in the STAT5 and Bcl-2 assays were on average < 60 yrs of age (STAT5: 55.8 (±9.9); Bcl-2: 47.1 (±13.5)) and impairments were not observed in the T_EMRA_ subset. While the average age of HCV^+^ individuals tested was higher than controls in most experiments, among HCV^+^ individuals, age was not associated with any differences. Lastly, while the CMV serostatus of our subjects was not determined, if there was active CMV infection in the HCV^+^ individuals, this would have been expected to reduce CD127 expression [78] and a difference in CD127 expression was not observed in most cases (Fig 1E and 1F). While CMV status and virus-specific cells do play a role in observed immune senescence and the decline of immune response in the elderly, and are numerous among circulating cells, we do not think this has significantly influenced the findings here.

In summary, CD8^+^ T-cells are phenotypically different and have impaired responsiveness to IL-7 in chronic HCV infection that is independent of CD127 expression and prevalent among bulk CD8^+^ T-cells. This dysfunction was particularly pronounced in the liver where high basal pSTAT5 levels could not be surmounted with added cytokine and basal Bcl-2 expression was significantly lower than in the blood. A consequence of progressive liver disease and generalized CD8^+^ T-cell dysfunction may result in insufficient responses to other concurrent infections, negatively influence vaccine immunogenicity (e.g. influenza, HBV) or contribute to the risk for hepatocellular carcinoma. Preliminary trials of T-cell mediated HCV vaccination of individuals with chronic HCV infection induced weaker T-cell responses than controls, suggesting that CD8^+^ cell dysfunction poses relevant challenges to the development of an effective therapeutic HCV vaccine [79]. Ongoing research may provide insights into the design of immune therapeutics for individuals whose cure rates with direct acting antiviral therapy are lower, such as those with advanced fibrosis, cirrhosis and those co-infected with HIV. Finally, HCV infection may be an important model of how liver disease affects CTL function, no matter its etiology, including nonalcoholic steatohepatitis and hepatocellular carcinoma.

## Supporting Information

S1 Fig Intrahepatic-CD8^+^ T-cells in HCV infection do not undergo further activation of STAT5 with γ_c_ cytokines past basal expression and have low basal Bcl-2 expression. (A) Blood-derived CD8^+^ T-cells and IH-lymphocytes from the same donors (n = 2) were cultured with STAT5-activating γ_c_ cytokines (IL-7 (0.1 or 1 ng/ml), IL-2 (100 ng/ml), or IL-15 (10 ng/ml)) and pSTAT5 expression (MFI). (B) Bcl-2 expression of unstimulated CD8^+^ T-cells was measured after overnight rest at 37°C (n = 1).
(TIF)

S1 File Table A-K. Raw data. (DOCX)
