# Supplementary figures and images for: Generalized Liver- and Blood-Derived CD8+ T-Cell Impairment in Response to Cytokines in Chronic Hepatitis C Virus Infection
Source: PLoS One. 2016 Jun 17;11(6):e0157055. doi: 10.1371/journal.pone.0157055 (PMC4912163; doi:10.1371/journal.pone.0157055)

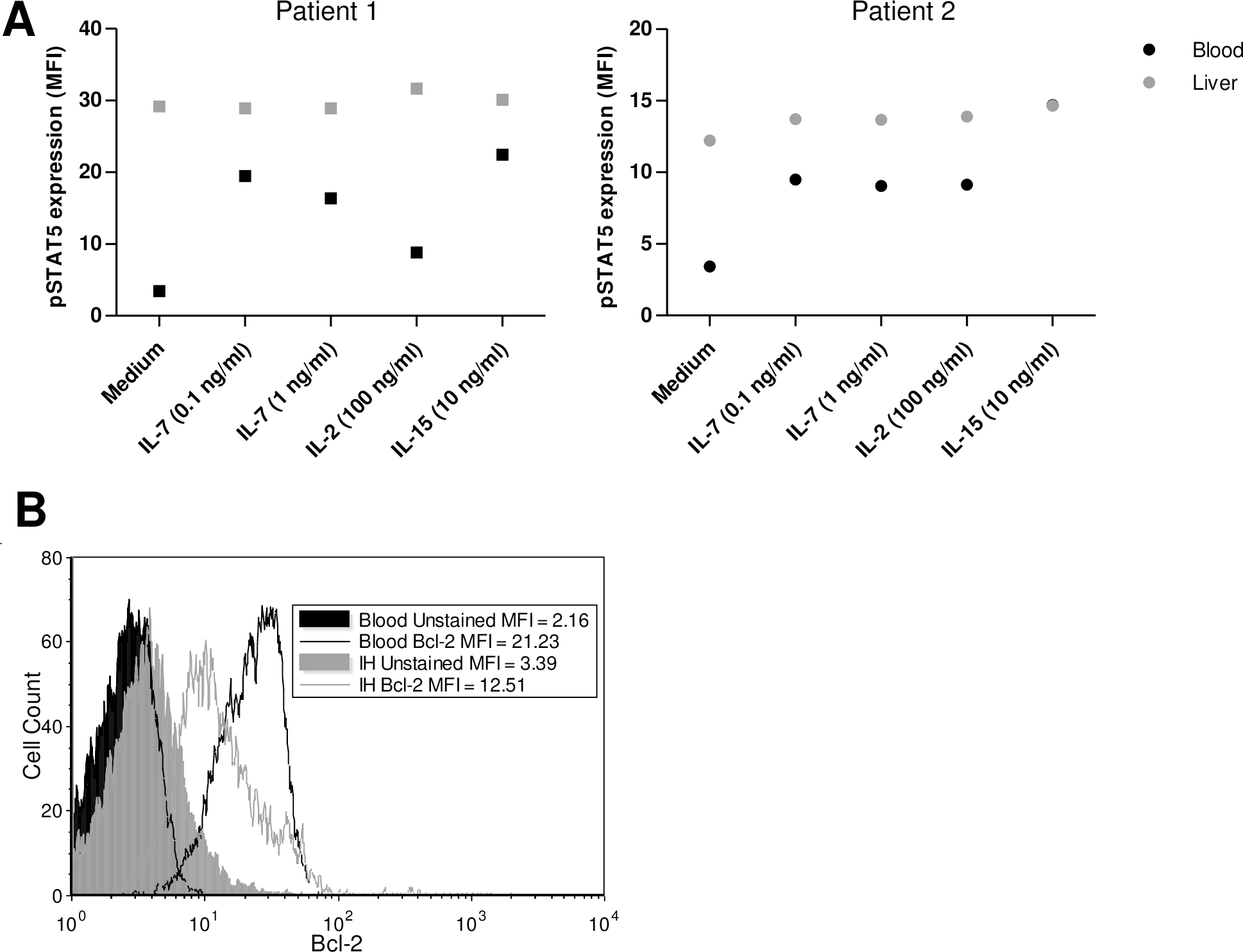

Supplement: S1 Fig — (A) Blood-derived CD8+ T-cells and IH-lymphocytes from the same donors (n = 2) were cultured with STAT5-activating γc cytokines (IL-7 (0.1 or 1 ng/ml), IL-2 (100 ng/ml), or IL-15 (10 ng/ml)) and pSTAT5 expression (MFI). (B) Bcl-2 expression of unstimulated CD8+ T-cells was measured after overnight rest at 37°C (n = 1). (TIF) [file pone.0157055.s001.tif]
