# Supplementary material for: Generalized Liver- and Blood-Derived CD8+ T-Cell Impairment in Response to Cytokines in Chronic Hepatitis C Virus Infection
Source: PLoS One. 2016 Jun 17;11(6):e0157055. doi: 10.1371/journal.pone.0157055 (PMC4912163; doi:10.1371/journal.pone.0157055)
Supplement: S1 File — (DOCX) [file pone.0157055.s002.docx]

**S1 File Table A. Subset distribution of CD8^+^ T-cells.**

| Status | % of CD8^+^ | | | |
| --- | --- | --- | --- | --- |
|  | Naïve | Central Memory | Effector Memory | EMRA |
| Control | 39.45 | 5.98 | 16.29 | 38.29 |
| Control | 34.56 | 8.68 | 26.66 | 30.01 |
| Control | 36.11 | 38 | 12.68 | 13.22 |
| Control | 46.91 | 31.88 | 8.02 | 13.2 |
| Control | 47.73 | 7.24 | 17.69 | 27.35 |
| Control | 33.36 | 8.13 | 23.55 | 34.96 |
| Control | 31.19 | 34.58 | 19.8 | 14.44 |
| Control | 38.72 | 20.82 | 15.56 | 24.9 |
| Control | 35.06 | 14.71 | 28.4 | 21.84 |
| Control | 27.53 | 12.95 | 32.51 | 27.02 |
| HCV | 32.95 | 17.34 | 18.33 | 31.38 |
| HCV | 17.44 | 26.51 | 40.13 | 15.93 |
| HCV | 28.37 | 42.01 | 14.35 | 15.27 |
| HCV | 24.21 | 32.26 | 33.29 | 10.25 |
| HCV | 35.53 | 26.51 | 22.04 | 15.91 |
| HCV | 9.51 | 9.52 | 60.16 | 20.18 |
| HCV | 13.92 | 34.82 | 26.63 | 24.63 |
| HCV | 36.75 | 10.57 | 30.09 | 22.6 |
| HCV | 30.47 | 20.22 | 26.81 | 22.5 |
| HCV | 28.5 | 9.77 | 25.82 | 35.91 |
| HCV | 32.89 | 35.64 | 14.97 | 16.51 |
| HCV | 30.01 | 16.92 | 33.84 | 19.23 |

**S1 File Table B. Expression of CD127 on CD8^+^ T-cell subsets.**

| Status | mCD127 Expression (%) | | | |
| --- | --- | --- | --- | --- |
|  | Naïve | Central Memory | Effector Memory | EMRA |
| Control | 62.34 | 65.78 | 44.84 | 12.62 |
| Control | 82.69 | 64.48 | 73.76 | 14.33 |
| Control | 72.64 | 49.17 | 17.15 | 4.87 |
| Control | 78.21 | 52.06 | 38.21 | 6.55 |
| Control | 63.36 | 67.87 | 17.47 | 12.91 |
| Control | 63 | 55.32 | 12.91 | 6.42 |
| Control | 81.17 | 54.39 | 33.09 | 6.22 |
| Control | 77.28 | 60.28 | 33.33 | 6.4 |
| Control | 82.69 | 68.63 | 32.48 | 11.61 |
| Control | 77.27 | 54.78 | 27.59 | 14.32 |
| HCV | 81.54 | 56.09 | 32.44 | 10.63 |
| HCV | 51.15 | 36.58 | 35.82 | 11.89 |
| HCV | 66.91 | 33.03 | 22.86 | 7.51 |
| HCV | 74.45 | 44.53 | 21.56 | 15.51 |
| HCV | 65.16 | 22.06 | 14.45 | 9.46 |
| HCV | 85.54 | 67.42 | 41.65 | 13.82 |
| HCV | 53.75 | 42.77 | 24.86 | 3.06 |
| HCV | 89.18 | 57.22 | 38.3 | 30.71 |
| HCV | 86.26 | 51.31 | 54.4 | 21.67 |
| HCV | 81.9 | 57.24 | 40.27 | 18.15 |
| HCV | 83.66 | 51.41 | 23.46 | 6.02 |
| HCV | 83.47 | 56.3 | 32.64 | 15.81 |

**S1 File Table C. Expression of pSTAT5 in response to IL-7.**

| Status | MFI | | | | | |
| --- | --- | --- | --- | --- | --- | --- |
|  | Medium | IL-7 (ng/ml) | | | | |
|  | 0 | 0.01 | 0.1 | 1 | 5 | 10 |
| Control | 5.56 | 5.8 | 6.17 | 11.59 |  | 17.1 |
| Control | 5.69 | 8.01 | 6.57 | 13.2 |  | 21.7 |
| Control | 4.32 | 7.84 | 15.28 | 14.07 |  | 13.97 |
| Control | 4.82 | 5.88 | 13.72 | 19.94 | 17.54 | 20.87 |
| Control | 3.63 | 5.38 | 9.95 | 20.76 | 21.74 | 21.33 |
| Control | 4.12 | 5.99 | 13.78 | 13.25 | 14.8 | 19.23 |
| Control | 3.44 | 5.26 | 10.7 | 12.47 | 11.24 | 11.18 |
| Control | 4.74 | 8 | 13.12 | 14.58 | 15.73 | 16.58 |
| Control | 3.25 | 5.29 | 10.65 | 11.89 | 12.19 | 10.81 |
| Control | 3.6 | 4.61 | 5.94 | 5.94 | 6.27 | 6.14 |
| Control | 5.64 | 4.35 | 7.05 | 8.34 | 11.52 | 10.72 |
| Control | 3.79 | 4.12 | 6.13 | 8.21 | 7.62 | 7.97 |
| HCV | 6.03 | 6.16 | 6.24 | 7.71 |  | 19 |
| HCV | 5.47 | 5.1 | 7.42 | 9.55 | 14 | 13.56 |
| HCV | 4.37 | 4.43 | 4.46 | 7.1 | 10.95 | 14.51 |
| HCV | 4.29 | 4.2 | 4.39 | 5.69 | 7.17 | 8.78 |
| HCV | 4.22 | 10.85 | 12.37 | 15.41 | 13.88 | 15.91 |
| HCV | 4.09 | 7 | 15.38 | 16.45 | 16.01 | 14.79 |
| HCV | 4.8 | 4.16 | 4.48 | 9.09 | 12.36 | 14.44 |
| HCV | 3.72 | 3.52 | 3.77 | 7.45 | 9.74 | 11.8 |
| HCV | 3.04 | 3.39 | 3.41 | 5.66 | 10.57 | 11.84 |

**S1 File Table D. Expression of pSTAT5 in response to IL-7 in CD8^+^ T-cell subsets.**

| Status | Naïve - MFI | | | | | Central Memory - MFI | | | | | |
| --- | --- | --- | --- | --- | --- | --- | --- | --- | --- | --- | --- |
|  | Medium | IL-7 (pg/ml) | | | | Medium | IL-7 (pg/ml) | | | | |
|  | 0 | 0.01 | 0.1 | 1 | 10 | 0 | 0.01 | | 0.1 | 1 | 10 |
| Control | 2.97 | 7.56 | 19.59 | 14.61 | 17.66 | 2.87 | 4.79 | | 14.08 | 13.2 | 13.96 |
| Control | 3.27 | 7.29 | 14.25 | 16.2 | 19.23 | 3.4 | 5.28 | | 11.51 | 12.1 | 14.37 |
| Control | 2.85 | 8.31 | 15.86 | 12.3 | 14.98 | 2.9 | 5.88 | | 12.32 | 10.48 | 13.32 |
| Control | 3.24 | 9.43 | 17.08 | 16.59 | 14.71 | 2.89 | 6.44 | | 13.25 | 13.71 | 11.47 |
| Control | 3.28 | 10.14 | 14.69 | 9.61 | 11.69 | 3.14 | 8.66 | | 15.02 | 9.18 | 10.85 |
| Control | 3.07 | 13.07 | 16.15 | 18.9 | 19.19 | 3.02 | 8.75 | | 13.66 | 14.22 | 14.18 |
| Control | 2.92 | 11.53 | 15.39 | 16.37 | 15.46 | 3.63 | 8.35 | | 10.04 | 10.22 | 11.04 |
| HCV | 2.88 | 10.15 | 13.9 | 10.53 | 9.84 | 3.09 | 8.33 | | 11.79 | 9.08 | 9.08 |
| HCV | 3.38 | 9.54 | 10.2 | 10.67 | 15.39 | 3.61 | 10.38 | | 10.48 | 11 | 14.94 |
| HCV | 2.96 | 4.39 | 6.04 | 5.99 | 6.3 | 2.88 | 3.76 | | 5.26 | 5.77 | 5.91 |
| HCV | 2.82 | 5.51 | 9.98 | 10.98 | 11.1 | 3.48 | 4.53 | | 8.28 | 9.07 | 9.7 |
| HCV | 3.05 | 5.4 | 7.88 | 8.46 | 10.3 | 3.05 | 5.25 | | 7.79 | 6.33 | 10.91 |
|  | Effector Memory - MFI | | | | | EMRA - MFI | | | | | |
|  | Medium | IL-7 (pg/ml) | | | | Medium | | IL-7 (pg/ml) | | | |
|  | 0 | 0.01 | 0.1 | 1 | 10 | 0 | | 0.01 | 0.1 | 1 | 10 |
|  | 2.85 | 3.78 | 9.06 | 8.34 | 9.67 | 2.86 | | 2.98 | 4.33 | 3.76 | 4.1 |
|  | 3.12 | 3.56 | 5.91 | 6.96 | 8.49 | 3.11 | | 3.11 | 3.47 | 3.77 | 4.14 |
|  | 3.3 | 5.03 | 10.44 | 9.11 | 11.24 | 3.53 | | 3.8 | 6.04 | 5.31 | 6.03 |
|  | 3.02 | 4.34 | 8.14 | 7.92 | 7.31 | 3.3 | | 3.43 | 4.35 | 4.31 | 4.11 |
|  | 3.2 | 6.37 | 11.66 | 7.8 | 9.43 | 3.36 | | 4.62 | 7.33 | 5.61 | 6.54 |
|  | 3.19 | 5.55 | 9.69 | 10.83 | 10.54 | 3.06 | | 3.54 | 4.33 | 4.8 | 4.82 |
|  | 2.59 | 5.66 | 7.79 | 8.53 | 7.93 | 2.55 | | 2.91 | 3.04 | 3.06 | 3.15 |
|  | 2.96 | 6.99 | 11.42 | 8.56 | 7.56 | 2.87 | | 4.62 | 5.77 | 4.94 | 4.03 |
|  | 3.39 | 6.64 | 7.92 | 7.7 | 9.78 | 3.46 | | 3.31 | 3.52 | 3.87 | 4.57 |
|  | 2.44 | 2.9 | 4.27 | 4.56 | 4.65 | 2.65 | | 2.99 | 3.68 | 3.82 | 3.87 |
|  | 3.08 | 3.75 | 6.82 | 7.84 | 8.26 | 2.71 | | 2.89 | 3.65 | 4.08 | 4.39 |
|  | 2.74 | 4.09 | 6.69 | 7.44 | 9.23 | 2.95 | | 3.83 | 5.61 | 6.23 | 7.42 |

**S1 File Table E. Ex vivo Bcl-2 expression of CD8^+^ T-cells.**

| Status |  | MFI |  |
| --- | --- | --- | --- |
|  | Unstained | Isotype | Baseline |
| Control | 2.47 | 3.96 | 22.35 |
| Control | 2.15 | 3.45 | 21.23 |
| Control | 2.73 | 5.27 | 25.22 |
| Control | 2.76 | 4.57 | 15.94 |
| HCV | 2.02 | 3.32 | 21.37 |
| HCV | 2.33 | 3.43 | 17.86 |
| HCV | 2.53 | 5.92 | 23.05 |
| HCV | 2.40 | 3.77 | 18.34 |
| HCV | 2.49 | 3.69 | 15.66 |

**S1 File Table F. Production of Bcl-2 in response to IL-7.**

| Status | MFI | | | | | |
| --- | --- | --- | --- | --- | --- | --- |
|  | Medium | IL-7 (pn/ml) | | | | |
|  |  | 0.01 | 0.1 | 1 | 5 | 10 |
| Control | 22.18 | 19.09 | 22.09 | 32.10 | 41.84 | 41.45 |
| Control | 18.41 | 19.62 | 23.91 | 30.56 | 35.01 | 34.40 |
| Control | 21.08 | 21.93 | 24.07 | 32.05 | 36.18 | 32.76 |
| Control | 16.08 | 18.16 | 19.88 | 27.92 | 33.95 | 33.64 |
| Control | 15.80 | 16.90 | 22.80 | 25.35 | 26.68 | 25.16 |
| Control | 18.95 | 18.67 | 25.30 | 32.25 | 32.13 | 34.42 |
| Control | 14.55 | 13.65 | 18.34 | 20.91 | 19.98 | 18.55 |
| Control | 16.63 | 17.99 | 23.04 | 28.76 | 27.56 | 30.61 |
| HCV | 14.1 | 16.6 | 22 | 23.6 | 27.7 | 28.11 |
| HCV | 17.07 | 16.23 | 18.23 | 19.53 | 22.59 | 25.17 |
| HCV | 22.33 | 13.87 | 14.6 | 17.93 | 24.42 | 21.82 |
| HCV | 23.38 | 14.66 | 15.75 | 19.11 | 20.5 | 23.58 |
| HCV | 16.53 | 17.24 | 17.67 | 24.47 | 23.5 | 27.16 |
| HCV | 14.76 | 15.29 | 18.55 | 23.97 | 24.35 | 24.07 |
| HCV | 20.11 | 21.14 | 19.8 | 22.24 | 29.33 | 30.28 |
| HCV | 15.28 | 13.9 | 18.17 | 23.84 | 27.73 | 25.71 |
| HCV | 17.48 | 18.42 | 25.4 | 38.65 | 36.24 | 40.11 |

**S1 File Table G. Proliferation of CD8^+^ T-cells in response to IL-7.**

| Status | PHA (0.2ug/ml) | IL-7 (10ng/ml) + PHA (0.2ug/ml) |
| --- | --- | --- |
| Control | 20.83 | 80.15 |
| Control | 9.21 | 62.30 |
| Control | 5.16 | 27.25 |
| Control | 10.25 | 34.47 |
| Control | 16.70 | 62.63 |
| Control | 10.78 | 46.12 |
| Control | 54.89 | 88.79 |
| Control | 25.50 | 61.22 |
| HCV | 30.28 | 71.47 |
| HCV | 21.7 | 79.94 |
| HCV | 8.62 | 50.89 |
| HCV | 19.93 | 55.87 |
| HCV | 36.32 | 57.74 |
| HCV | 33.59 | 60.04 |
| HCV | 15.19 | 43.22 |
| HCV | 9.28 | 44.6 |

**S1 File Table H. Subsets distribution of CD8^+^ T-cells from liver biopsied HCV^+^ individuals.**

| Location | % of CD8^+^ | | | |
| --- | --- | --- | --- | --- |
|  | Naïve | Central Memory | Effector Memory | EMRA |
| Blood (A) | 32.95 | 17.34 | 18.33 | 31.38 |
| Liver (A) | 11.64 | 38.93 | 39.62 | 9.81 |
| Blood (B) | 17.44 | 26.51 | 40.13 | 15.93 |
| Liver (B) | 10.71 | 35.71 | 50.12 | 3.46 |
| Blood (C) | 35.53 | 26.51 | 22.04 | 15.19 |
| Liver (C) | 18.82 | 35.0 | 36.95 | 9.24 |
| Liver (D) | 3.70 | 46.64 | 43.85 | 5.81 |

**S1 File Table I. Expression of CD127 on CD8^+^ T-cells from liver biopsied HCV^+^ individuals.**

| Location | mCD127 expression (%) | | | |
| --- | --- | --- | --- | --- |
|  | Naïve | Central Memory | Effector Memory | EMRA |
| Blood (A) | 81.54 | 56.09 | 32.44 | 10.63 |
| Liver (A) | 92.07 | 63.65 | 35.38 | 33.70 |
| Blood (B) | 51.15 | 36.58 | 35.82 | 11.89 |
| Liver (B) | 94.36 | 43.50 | 15.08 | 31.03 |
| Blood (C) | 65.16 | 22.06 | 14.45 | 9.46 |
| Liver (C) | 66.82 | 12.29 | 5.79 | 19.65 |

**S1 File Table J. Expression of pSTAT5 in CD8^+^ T-cells from liver biopsied HCV^+^ individuals.**

| Location | Medium | IL-7 (0.1ng/ml) | IL-7 (1ng/ml) | IL-2 (100ng/ml) | IL-15 (10ng/ml) |
| --- | --- | --- | --- | --- | --- |
| Blood (A) | 3.46 | 19.49 | 16.4 | 8.83 | 22.46 |
| Liver (A) | 29.16 | 28.89 | 28.92 | 31.65 | 30.09 |
| Blood (B) | 3.42 | 9.48 | 9.05 | 9.13 | 14.68 |
| Liver (B) | 12.21 | 13.71 | 13.66 | 13.89 | 14.64 |

**S1 File Table K. Expression of basal Bcl-2 in CD8^+^ T-cells from liver biopsied HCV^+^ individuals**

| Location | MFI | |
| --- | --- | --- |
|  | Unstained | Baseline |
| Blood | 2.02 | 21.37 |
| Blood | 2.33 | 17.86 |
| Blood | 2.53 | 23.05 |
| Blood | 2.40 | 18.34 |
| Blood | 2.49 | 15.66 |
| Liver | 3.47 | 14.04 |
